# Development and implementation of a comprehensive ultrasound curriculum for medical students: The Bonn internship point-of-care-ultrasound curriculum (BI-POCUS)

**DOI:** 10.3389/fmed.2023.1072326

**Published:** 2023-03-24

**Authors:** Florian Recker, Valentin Sebastian Schäfer, Wolfgang Holzgreve, Peter Brossart, Simon Petzinna

**Affiliations:** ^1^Department of Obstetrics and Prenatal Medicine, University Hospital Bonn, Bonn, Germany; ^2^Clinic of Internal Medicine III, Oncology, Hematology, Rheumatology, and Clinical Immunology, University Hospital Bonn, Bonn, Germany; ^3^University Hospital Bonn, Bonn, Germany

**Keywords:** point-of-care-ultrasound, ultrasound curriculum, curriculum, development, medical students

## Abstract

**Background:**

Point-of-care ultrasound (POCUS) is rapidly gaining ground within different areas of applications. Despite the high and increasing relevance of ultrasound, the availability of structured training programs in medical schools is still limited. Therefore, many doctors keep acquiring all their ultrasound skills throughout their postgraduate training. As a result, new residents lack theoretical and practical ultrasound abilities that are critical in everyday clinical practice. In order to improve this, we created and implemented a complete ultrasound curriculum for all medical students throughout their internship year that focuses on hands-on abilities in ultrasound imaging.

**Methods:**

We used Kern‘s six-step model of curricular development comprising (1) problem identification and general needs assessment, (2) needs assessment of the targeted learners, (3) goals and objectives, (4) educational strategies, (5) implementation, and (6) evaluation and feedback by board-certified ultrasound experts. A two rounds Delphi process with multilevel, self-completed questionnaires and individual using a 9-point Likert scale and free text comments was used to identify learning objectives and reach agreement on the content of the curriculum.

**Results:**

The curriculum developed is aimed at students with no or little experience in their internship year and will be taught as part of their weekly-based internship training courses consisting of 2 hours of theory and 3 hours of practical training. The training will be conducted within a modular framework focusing on the key requirements of POCUS with increasing levels of complexity in accordance with the recommendations of the German Society for Ultrasound in Medicine (DEGUM), the European Federation of Societies for ultrasound in Medicine and Biology (EFSUMB) and the World Federation for ultrasound in Medicine and Biology (WFUMB). A longitudinal e-learning system will be implemented in addition to the practical and theoretical teaching units to track and examine the progress of the students.

**Conclusion:**

Early integration of ultrasound training into medical education as part of a structured and standardized broad ultrasound curriculum enables medical students to acquire basic skills and apply them practically. Fundamental scanning skills are acquired by hands-on exercises in small, supervised groups as part of BI-POCUS. BI-POCUS therefore provides an excellent opportunity to improve the clinical skills of future physicians. More research is needed to analyze the learning outcomes for medical students and the improvement of the patient’s outcome by establishing such an ultrasound curriculum.

## Background

In recent years, the importance of ultrasound in medicine has increased substantially, owing to its diverse advantages as an imaging modality. In contrast to competing imaging techniques, such as computer tomography (CT) or magnetic resonance tomography (MRI), ultrasound offers superior mobility, speed, and availability. Additionally, it is an extremely safe and non-invasive technique for real-time visualization of the human body (1), and can be effectively employed for guiding interventional procedures. As a result, ultrasound is rapidly evolving into a fundamental diagnostic tool across various medical specialties, and has, in fact, become the most commonly utilized imaging tool in clinical practice (2, 3). Widespread clinical application of ultrasound holds potential for promoting interdisciplinary integration and enabling an intensified interdisciplinary treatment approach (4).

Point-of-care ultrasound (POCUS) is gaining significant traction across a diverse range of applications. It can be performed using either a conventional ultrasound device or a portable/hand-held device. As a result of these technological advancements, ultrasound diagnostic equipment has become significantly more affordable (5, 6). Although hand-held devices currently vary in the quality of images generated, they serve a specific purpose based on the area of application. The ability to rapidly visualize and address clinical concerns at the bedside is increasingly vital. Bedside utilization of POCUS offers potentially life-saving insights regarding the cardiac status, presence of free abdominal fluid, and the occurrence of a pneumothorax with a high degree of accuracy (7). Furthermore, POCUS has the potential to enhance the quality of physical examinations, confirm the physician’s preliminary clinical observations, and ultimately improve patient outcomes (8).

Compared to other imaging modalities, sonographic diagnosis partially relies on the skills of the operator. Performing an ultrasound scan requires a blend of theoretical physical knowledge, anatomical proficiency, pathology identification, and the ability to manipulate the ultrasound device to generate precise images. In addition, expertise in pathophysiology and spatial vision is critical. Fundamental skills are often acquired through supervised clinical training, followed by independent practice (9, 10). Due to advancements in technical capabilities, expanding areas of application, and deepening understanding of ultrasound imaging, a continuous learning process is imperative. Failure to maintain up-to-date knowledge and expertise may result in misinterpretations, errors, and poor reproducibility of equipment usage (11). Various organizations worldwide are working to implement standardized ultrasound education. The World Federation for Ultrasound in Medicine and Biology (WFUMB), the European Federation of Societies for Ultrasound in Medicine and Biology (EFSUMB), and the German Society for Ultrasound in Medicine (DEGUM) are all dedicated to promoting and standardizing ultrasound imaging (12).

Despite the growing significance of ultrasound, structured training programs for medical students remain considerably limited. Consequently, newly-minted residents often lack the theoretical and practical ultrasound skills that are urgently required in daily clinical practice. Many physicians acquire their ultrasound skills exclusively through postgraduate training. In response, several universities have successfully offered ultrasound education for several decades, utilizing a range of different approaches, teaching methods, and curricula (8, 12–15). Recent studies have evaluated the implementation of ultrasound educational curricula in both graduate and postgraduate programs (15–22), testing practical training in small groups, online training, and case-based discussions (23). These studies have illuminated various aspects of the implementation of medical ultrasound education, ranging from strictly subspecialty-focused programs to more general ultrasound standards training. Continuous performance assessment of sonoanatomical and ultrasound-related clinical knowledge can increase learning success and ultimately enhance clinical practice outcomes (3). Integrating ultrasound education into existing coursework may also support successful implementation and bolster general knowledge of anatomy, physiology, and pathology (15).

However, various problems became evident during attempts to implement ultrasound education. Training in ultrasound necessitates practical instruction to ensure competency. Success is more likely when the group size is smaller and the tutor can engage more intensively with the trainees. However, key limiting factors include resources in terms of time, personnel, and equipment (19, 24–28). Several studies have attempted to address this issue by implementing a peer-teaching process (24). Interestingly, not only could non-inferiority be demonstrated with regard to peer-teaching versus classical teaching approaches for trainees’ benefit, but even the peer-teachers themselves can benefit from this educational approach (18, 29).

This study represents the first attempt to address the numerous challenges associated with developing a comprehensive, structured, and reproducible ultrasound education curriculum (30). BiPOCUS, designed to align with international and national ultrasound guidelines, is a universally implementable, competency-based, and quality-driven curriculum tailored to undergraduate students in their internship year, rooted in internationally peer-reviewed ultrasound standards. BiPOCUS incorporates essential basic skills and recent innovations, such as hand-held ultrasound devices, in an innovative educational process. As a result, students are provided with a diversified ultrasound education that serves as an essential platform for continued skill development throughout their residency.

## Methods

Our objective was to develop a highly advanced, universally implementable, and reproducible POCUS curriculum, utilizing Kern’s six-step approach, for undergraduate students in their mandatory internship year within the German medical curriculum (31). During this year, students acquire practical skills and expand upon previously obtained knowledge through hands-on patient practice. Given the distinct lack of ultrasound training within the curriculum thus far, most medical students possess only rudimentary ultrasound training and may be considered beginners in this field. In order to comprehensively address this issue, we employed Kern’s six-step approach to problem identification, needs assessment of targeted learners, goal and objective setting, educational strategy formulation, implementation, and evaluation and feedback.

### Problem identification

To determine the necessary content for a comprehensive ultrasound education for undergraduate medical students during their internship year, we conducted a thorough review of existing literature and frameworks. The educational objectives for students in their internship year are based on the guidelines for the “Basic Course in Ultrasound of the Abdomen and Retroperitoneum,” the learning objectives of the emergency ultrasound group of DEGUM and EFSUMB, as well as the student medical education guidelines of WFUMB. These guidelines were developed and adopted in collaboration with the Societies for Internal Medicine, Surgery, and Radiology, as well as the task force for emergency medicine. We also reviewed complementary published literature to identify critical ultrasound skills or algorithms for medical students and residents across various specialties, ensuring a universally implementable and reproducible curriculum for internship year students that is based on international peer-reviewed quality standards.

### Needs assessment of the targeted learners

We ensured alignment with the competencies proposed by DEGUM/EFSUMB/Abdominal Ultrasound, as well as established ultrasound projects. The various aspects of ultrasound identified in our assessment, along with identified application areas, techniques, and implementations, were thoroughly discussed with members of different faculties at the University of Bonn. To ensure adequate needs assessment of targeted learners, we conducted a survey with 1,040 medical students from 31 universities in Germany within our research group (32). Although a high level of interest in ultrasound education and affiliated peer teaching was revealed, students reported insufficient time allotted for ultrasound education in the curriculum, as well as a lack of courses offered by medical schools. These results were taken into consideration during the implementation of BIPOCUS.

### Goals and objectives

**Figure 1:**
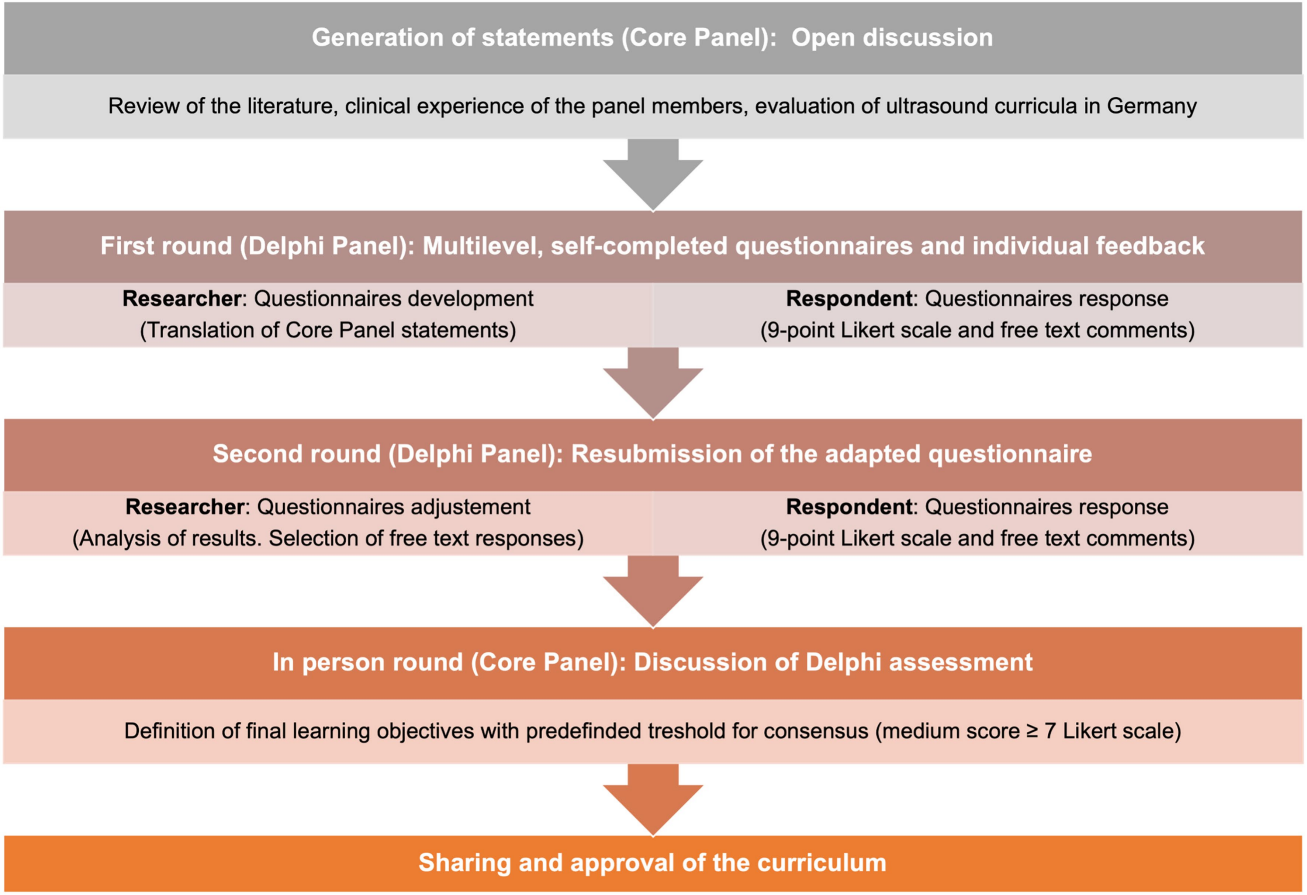
Needs assessment and Delphi process. Illustration of the steps of the Delphi process in the context of the needs assessment.

Each identified ultrasound technique, application area, and implementation was operationalized with consideration given to their technical, physical, and pathologic characteristics. The Delphi process involved clinical members with EFSUMB and DEGUM Level I-III certifications from various specialties and medical students. The Delphi technique is a consensus method used in research for problem-solving, concept generation, or priority definition (33). In our study, the Delphi process was employed to identify learning objectives and reach an agreement on the content of the curriculum. Initial statements were generated through open discussion within a Core Panel consisting of the author of this study, and a total of 10 members participated in two rounds of Delphi using multilevel, self-completed questionnaires and individual feedback. Participants expressed their agreement or disagreement using a 9-point Likert scale and free text comments. The statements of the questionnaires were generated based on literature, clinical experience of panel members, and previous evaluation of ultrasound curricula in Germany by a Core Group within our study group (32). Following the first Delphi process, the data was analyzed, and a new questionnaire, including selected free text responses, was created. Consensus was defined as a medium score greater than or equal to 7, as previously described (Figure 1) (33). Throughout the process of establishing the guidelines, faculty members from specialties that provided the services and medical students played vital roles.

### Educational strategies

The implemented educational strategies needed to cover a range of requirements to address all areas and aspects of the curriculum. Consequently, we combined different educational strategies to ensure the teaching of both practical skills and theoretical knowledge. Specifically, we focused on the techniques developed, used, and evaluated by DEGUM and EFSUMB for ultrasound medical student training. Theoretical knowledge was delivered through traditional educational strategies, such as lectures and manuscripts, while practical skills were developed through tutor-supervised training sessions. To categorize the level of competence required, we categorized the subsidiary components into hierarchical categories with ascending degrees of complexity. Since the learning goals comprised both theoretical knowledge and practical skills, different educational strategies were blended to cover all areas and aspects of the curriculum. For ultrasound training, we used the techniques developed, applied, and evaluated by DEGUM and EFSUMB, including scripts and lectures for theoretical knowledge and supervised hands-on training for practical application of the knowledge.

### Implementation

An appropriate time slot was chosen for the implementation of the BIPOCUS curriculum in educational and clinical settings. To avoid implementation problems, the teachers, who were board-certified ultrasound experts, were involved in the development and implementation of the curriculum and had already been part of ultrasound teaching programs. Additionally, the development process was guided by award-winning didactics experts who ensured adequate preparation for the curriculum’s requirements for all tutors. As a result, most of the necessary infrastructure for organizing the ultrasound curriculum was already established before the implementation stage.

### Evaluation and feedback

Finding the time and resources for ultrasound training programs within an already overloaded curriculum can be a challenge. Early research has shown that medical students can acquire the psychomotor and interpretive abilities necessary for effective focused ultrasound in small groups. For example, first-year medical students were able to effectively use portable ultrasound following six 90 min courses on abdominal, cardiovascular, genitourinary, and musculoskeletal applications. The WFUMB and EFSUMB have recently devised strategies to achieve this objective within European medical institutions (12). The BI-POCUS curriculum incorporates a multi-formative assessment and corresponding feedback, including the concepts of adaptive testing in the ultrasound teaching process. The corresponding feedback occurs through a global practical assessment, as well as learning objective-specific assessments, which utilize repetitive, adaptive testing strategies. Additionally, general feedback is obtained from students *via* questionnaires. The presented concept is constantly evolving at the level of evaluation and feedback and is accompanied by the Centre for Evaluation and Methodology at the University of Bonn.

## Results

We have developed BiPOCUS, an ultrasound curriculum that meets the needs of undergraduate students with regard to the growing demand for ultrasound imaging. The reproducible, competency-based, and quality-oriented nature of BiPOCUS is ensured through its alignment with international and national guidelines, involvement of board-certified ultrasound experts, and collaboration with award-winning didactic specialists. As such, BiPOCUS is not intended as a location-specific enhancement of education, but rather as a universally implementable curriculum for internship year students that adheres to international peer-reviewed quality standards.

### Problem identification

There are several studies published dealing with the requirements to achieve basic quality standards in ultrasound imaging (8, 12, 15, 34). It is expected that all medical students receive at least basic theoretical and technical training in ultrasound during their internship year. This is especially important as ultrasound imaging, and POCUS in particular, is gaining popularity for various applications. The dynamic and real-time capabilities of ultrasound are well-suited for clinical examination and interpretation by physicians, enabling rapid and appropriate treatment decisions. The early implementation of POCUS into the curriculum can have a significant impact on the quality of future examinations by young physicians. However, there is currently no standardized ultrasound curriculum integrated into the medical school curriculum in Germany. As a result, there is a strong demand for systematic teaching of basic ultrasound skills (32). Additionally, students face structural problems, such as a lack of ultrasound equipment and rooms, a shortage of qualified training staff, and limited time within the curriculum due to the lack of standardized implementation.

### Needs assessment of the targeted learners

**Figure 2:**
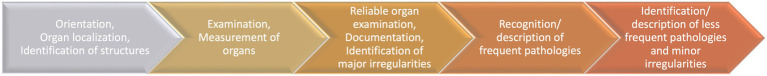
Degrees of complexity. Five-step approach for the acquisition of ultrasound skills with ascending degrees of complexity.

To assess the practical ultrasound skills of students, a five-step approach was developed with increasing levels of complexity. The fifth level aims to provide competencies required for independent operation during 24 h shifts in the emergency room or intensive care unit, and in residency. The practical ultrasound skills were categorized into the following levels (see Figure 2).

As prior theoretical knowledge is usually required for performing hands-on skills, these were also defined. The levels of competency taught to students substantially equal Level I/ II, with a maximum of Level III in certain sub competencies. Therefore, only levels I-IIl were described in more detail (Supplementary Table S1).

### Goals and objectives

**Figure 3:**
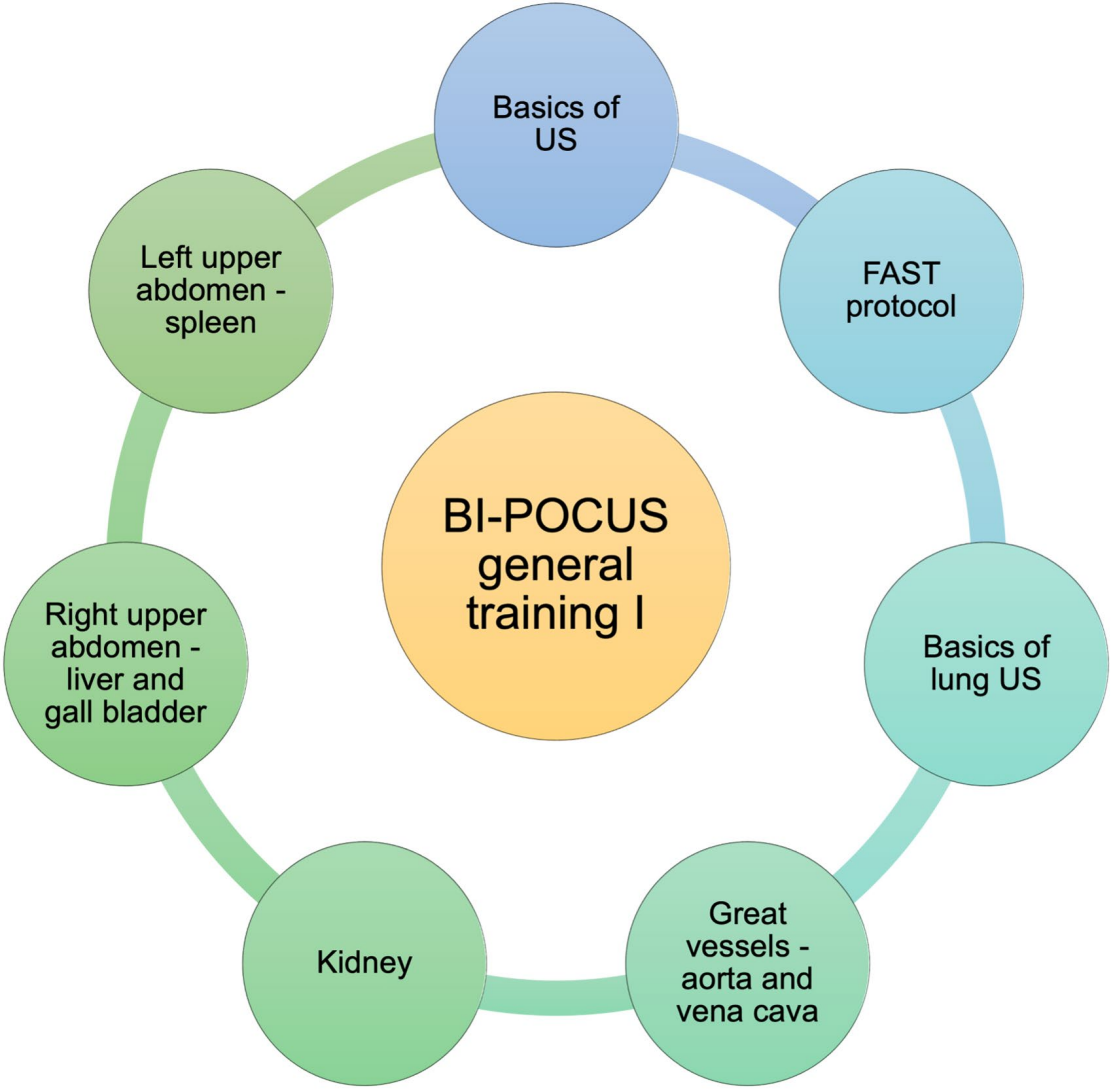
BI-POCUS – general training, educational content (Module 1). Modular framework with subdivision of the learning objectives included in the first module of the BI-POCUS curriculum; abbrev.: Ultrasound (US), focused assessment with sonography for trauma (FAST).

**Figure 4:**
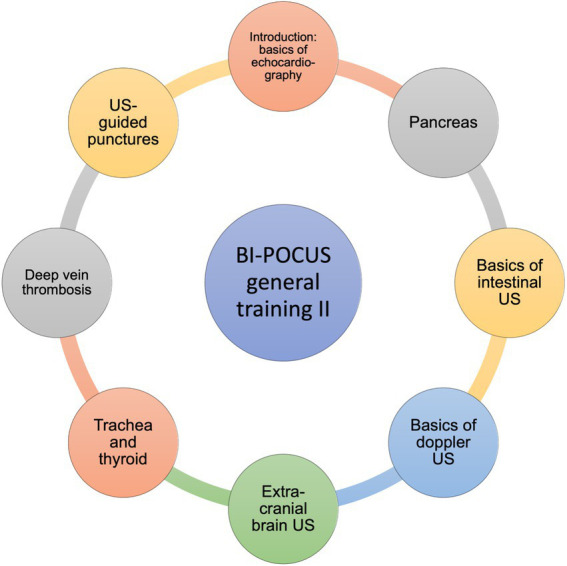
BI-POCUS – general training II, educational content (Module 2). Modular framework with subdivision of the learning objectives included in the second module of the BI-POCUS curriculum; abbrev.: Ultrasound (US).

**Figure 5:**
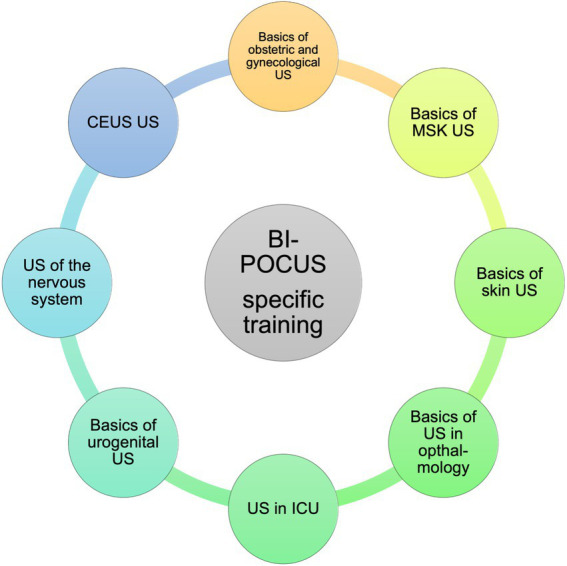
BI-POCUS – specific training, educational content (Module 3). Modular framework with subdivision of the learning objectives included in the third module of the BI-POCUS curriculum, abbrev.: Ultrasound (US), musculoskeletal (MSK), intensive care unit (ICU), contrast enhanced ultrasound (CEUS).

The needs assessment resulted in the implementation of the subsequent modular framework, focusing on essential requirements of POCUS (Figures 3–5). Students are able to further develop their theoretical and practical skills in weekly courses. The following goals and objectives for medical students could be identified (Table 1).

**Table 1:** BI-POCUS competence levels.

Topic	Lesson (method)	Level 1 objectives	Level 2 objectives	Level 3 objectives	Assessement of competency
Technical basics	Theory (CES)	1) Explain basic principles for image generation and frequency range of ultrasound. 2) Identify different transducers and their areas of application 3) Apply basic ultrasound-specific terminology 4) Identify artifacts (sound shadows, dorsal sound amplification)	1) Explain A, B, M-mode; artifacts part II	1) Explain and apply tissue Harmonic Imaging	Multiple-choice exams
	Practise (TSTS)	1) Select transducers problem based 2) Apply ultrasound machine setting (gain, penetration depth, image and loop saving)	1) Apply device functions: Focus, Frequency, Time-Gain Compensation, Penetration Depth		OSCE/ DOPS
Patient Management	Theory (CES)	1) Assess patient sobriety 2) Assess medical history		1) Explain specifics of the examination of unconscious patients, children, infants	Multiple-choice exams
	Practise (TSTS)	1) Demonstrate a respiratory maneuver 2) Demonstrate positioning of patient 3) Demonstrate communication throughout examination	1) Handle unusual patient positioning	1) Handle of communication with anxious patients	OSCE/ DOPS
Transducer handling	Theory (CES)	1) Explain abdominal sectioning	1) Vary conventional diagonal imaging		Multiple-choice exams
	Practise (TSTS)	1) Apply transducer positioning 2) Orientate 3) Vary pressure application			OSCE/ DOPS
Preparation	Theory (CES)	1) Explain indications and limitations of abdominal ultrasound examination			Multiple-choice exams
	Practise (TSTS)	1) Apply optimal ultrasound conditions 2) Demonstrate positioning of the ultrasound device			OSCE/ DOPS
Abdominal vessels	Theory (CES)			1) Explain variations of the arterial vascular supply	Multiple-choice exams
	Practise (TSTS)	1) Identify and localize the following structures: abdominal aorta, coeliac trunc, superior mesenteric artery, caval vein, portal vein, lienal vein, renal artery and vein, iliacal artery and vein 2) Display in longitudinal and cross-sectional images	1) Identify and localize the following vessels: Inferior mesenteric artery, common hepatic artery, lienal artery; 2) Perform the vena cava collapse test 3) Display in longitudinal and cross-sectional image	1) Apply Doppler sonography of the portal vein and renal artery	OSCE/ DOPS
Pancreas	Practise (TSTS)	1) Explain pancreatic parts 2) Identify and demonstrate lead structures	1) Identify and localize: the common hepatic duct (DHC) 2) Optimize image in difficult examination conditions 3) Apply translienal pancreatic imaging	1) Identify pancreatic duct and variants (uncinate process)	OSCE/ DOPS
Liver	Practise (TSTS)	1) Localize and demonstrate the right and left liver sections 2) Compare of echogenic differences (liver and kidney) 3) Localize the portal vein in the hepatic hilum 4) Visualize the inferior caval vein junction 5) Identify and localize the caudate lobe	1) Visualize and demonstrate hepatic vein – portal vein	1) Screen the liver segments 2) Assess liver transcostal	OSCE/ DOPS
Biliary ducts/ vessels	Practise (TSTS)	1) Localize and demonstrate the gallbladder subcostally 2) Assess gall bladder filling status	1) Assess the gallbladder wall 2) Visualize and demonstrate of hepatic propria artery and DHC 3) Identificate fissura interlobularis	1) Detect postprandial gallbladder 2) Track intrahepatic bile duct 3) Demonstrate stone criteria	OSCE/ DOPS
Kidney, adrenal gland	Practise (TSTS)	1) Localize the kidneys bilaterally 2) Identify renal parenchyma and renal medulla	1) Visualize renal hilus 2) Visualize and measure of organ in its largest diameter	1) Demonstrate variants 2) Demonstrate sonomorphology of renal cysts and renal stones 3) Identify adrenal lobe	OSCE/ DOPS
Spleen	Practise (TSTS)	1) Localize the organ	1) Visualize Hilar 2) Measure of length and depth	1) Identify secondary spleen	OSCE/ DOPS
Small pelvis	Practise (TSTS)	1) Localize and demonstrate urinary bladder, prostate/ uterus	1) Asssess urinary bladder volume	1) Demonstrate jet phenomenon	OSCE/ DOPS
Thyroid gland	Practise (TSTS)	1) Localize and demonstrate the right and left lobes and isthmus.	1) Assess volume		OSCE/ DOPS
Lymph nodes	Practise (TSTS)	1) Explain the physiological sonomorphology of lymph nodes	1) Explain major abdominal lymph nodes, neck lymph nodes, malignancy criteria.	1) Demonstrate the level assignment of the cervical lymph nodes	OSCE/ DOPS
Focused assessment with sonography for trauma (FAST)	Practise (TSTS)	1) Assess of fluid in the following areas: pericardial, right and left pleural cavity, Morison’s pouch, Koller’s pouch, rectovesical excavation / Douglas space			OSCE/ DOPS
Duplex sonography	Practise (TSTS)	1) Explain basics of the Doppler effect and its applications 2) Localize and demonstrate direction of flow	1) Explain pulse repititon frequency	1) Assess resistance index/ Flow profile	OSCE/ DOPS
Vessels	Practise (TSTS)	1) Localize and demonstrate carotid artery with internal and external branches		1) Demonstrate deep leg veins	OSCE/ DOPS

Topics and competence levels of the individual organ-related teaching units in the BI-POCUS curriculum. TSTS, tutor-supervised training sessions; CES, classical educational strategies (lectures and manuscripts); OSCE, Objective Structured Clinical Examination; DOPS, Direct Observation of Procedural Skills.

#### Module 1: the general part of the curriculum I

Students complete a basic educational program throughout their internship. The latter focuses on the basic knowledge of general emergency protocols, which every undergraduate student should acquire by the end of the internship year. We hereby focused on the essentials of the global valid standard protocols of the respective professional societies.

#### Module 2: the general part of the curriculum II

The second general element of the course emphasizes more specific areas. However, these areas are important aspects of general ultrasound education. They should be well understood by all graduates. More attention is given to clinical integration. Therefore module 1, it’s educational content and skills, are obligatory. The second general topic complex also includes issues addressed in the advanced courses of the DEGUM and EFSUMB. These are integrated complementarily into the education of the medical students.

#### Module 3: the subject specific part of the curriculum

After successful completion of the two general parts of the curriculum, the next module is focused on the subject specific areas. These specific areas can be chosen by the students. However, if interested, all subject specific subjects can be covered, allowing the graduate to choose a clinical track into each subject and to experience and learn about the subject specific integration of sonography. The individual subject specific areas of BI-POCUS were designed as shown below. These may also be supplemented by further specific subjects in the future.

### Educational strategies

Learning ultrasound is very different from many other clinical jobs that may be performed in the real world with a simple method and predictable results. But operating an ultrasound probe and setting up the device requires both technical know-how and the ability to think critically and hunt for fresh information in the ultrasound image. Because it is a diagnostic procedure, it cannot yield a result that can be predicted (35). A popular and effective approach of teaching ultrasound is through brief didactic modules followed by hands-on activities during small group sessions (12, 35, 36). However, many publishers are considering and utilizing alternative tactics. Cartier et al. used a learning theory approach to design an ultrasound course for medical students that includes cognitive, behavioral, and constructivist learning components (35). The EFSUMB recommends a constructivist approach to teaching during pre-clinical training, where knowledge is taught through organ- and topic-specific modules that also incorporate clinical aspects.

To focus on the practical skill of ultrasound, we deliberately excluded the treatment of certain illnesses to a significant extent. Only a few disorders are taught to provide a better understanding of unique situations and ultrasound findings, but these are not the main areas of interest for BI-POCUS. The main objective was to set a standard for uniform basic ultrasound training within the BI-POCUS curriculum.

For this purpose, the BI-POCUS curriculum is structured as a three-level program based on abdominal ultrasound protocols. By the end of the program, students are expected to achieve levels I and II in all areas, with level III being covered in some areas. However, level III is designed as a transition to additional medical education and cannot be fully attained within the BI-POCUS program. Nevertheless, this degree of ability is also demonstrated, ensuring that students gain some of the necessary skills in this area as part of their BI-POCUS training. A detailed breakdown of the individual competence levels is provided in the following table (Supplementary Table S1). Ultrasound training is carried out in an interdisciplinary manner across both study sections to ensure good integration and implementation in the curriculum of medical degrees.

### Implementation

The BI-POCUS Curriculum was introduced by the Medical Faculty of Bonn in 2022, with the optimal implementation time slot identified during weekly-based internship training courses. The relevant faculty members and key stakeholders were involved from the project’s inception and supported the curriculum. A corresponding expert group was established, consisting of various individual members of the sonography departments, and the necessary infrastructure to support the curriculum was subsequently developed. Additionally, a dedicated working group has been formed to provide long-term assistance for this tutor-based longitudinal initiative. Over 70% of the entire course is composed of practical content offered in different course forms, with appropriate DEGUM-certified tutors. Throughout the course, teachers provide practical training in small groups of three to four students, with each participant directly discussing the images and section planes they created during the session. Transducer guidance is altered if necessary, and the participants practice the sectional planes in line with the guidance of DEGUM, EFSUMB, and WFUMB. The practical components of each session last five to six hours, allowing each participant ample time to practice practical ultrasonography skills once a week. All participants are college students in their internship year, the majority of whom have had little to no previous ultrasound experience.

### Evaluation and feedback

**Figure 6:**
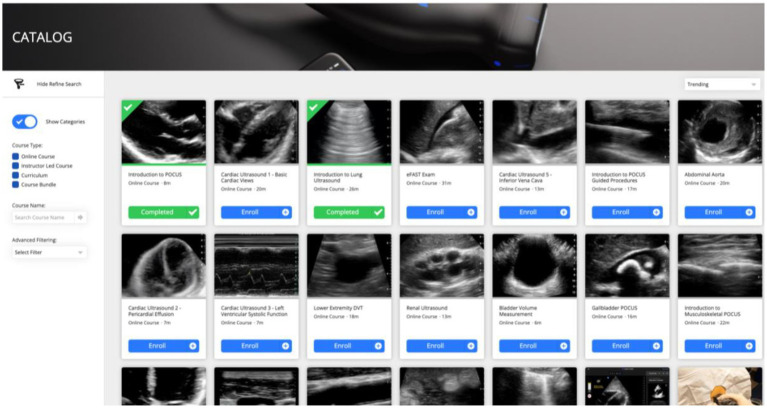
BI-POCUS – e-learning System. The integrative e-learning system with variable and topic-specific modules will be implemented in complement to the BI-POCUS curriculum, and will be adapted to the individual needs and abilities of each participant.

Within the BI-POCUS curriculum, a longitudinal e-learning system was implemented alongside the practical and theoretical teaching units to monitor and evaluate the students’ progress. The ButterflyAcademy^®^ was integrated into the curriculum, which provides individual learning units or topic modules that are accessible to students. The learning progress is also evaluated through individual tests at the end of each module, consisting of theoretical parts (multiple-choice exams) and practical parts (OSCE and DOPS), complemented by general learning surveys and participant feedback surveys. The corresponding examination formats and their integration within the constructive alignment of the curriculum are presented in Table 1. The individual formats, including multiple-choice, OSCE, and DOPS, illustrate the different examination taxonomies, which are further supported by the adaptive e-learning system (see Figure 6).

In addition, BI-POCUS includes individually adaptive computer-based training for image recognition and interpretation to improve the accuracy of diagnosis in POCUS.

Therefore, the image recognition and interpretation training, which aims to deepen theoretical knowledge, plays an important role. An individualized BIT, which is focused on the user’s skills and abilities, represents a new and innovative approach to practicing these critical skills in a more targeted and focused manner, thereby improving diagnostics in terms of reliability and efficiency. Additionally, scientific projects in the field of automated image assessment have been initiated in collaboration with the Department of Artificial Intelligence in Sectional Imaging, leading to the implementation of a completely new assessment tool in BI-POCUS.

In evaluating BI-POCUS, it is necessary to consider two aspects: assessing the diagnostic competence of the trainees upon completion of the curriculum and evaluating the effectiveness of the curriculum by monitoring the development of the competence levels throughout BI-POCUS. To achieve this, a reliable and valid test is used that is based on both global and direct objective checklist ratings. The assessment procedure is directly based on the trainees’ competency and is supplemented by general and topic-specific questionnaires that are completed by the respective students.

The students who participate in BI-POCUS receive a customized e-learning concept that is tailored to their individual needs. In the future, tele-didactic elements will also be integrated into the practical hands-on course to enhance the overall learning experience.

## Discussion

The objective of this study was to develop a highly adaptive Point-of-Care Ultrasound (POCUS) curriculum that meets the requirements of medical schools and prepares their students for future residency. Currently, ultrasound is only offered to a limited extent at Bonn University Hospital as a voluntary course (32). It has not been systematically integrated into basic medical training for all students. To address this gap, we have designed a comprehensive curriculum that equips all medical students in their internship year with practical knowledge and basic skills focused on point-of-care applications. Our program, BI-POCUS, introduces POCUS into clinical routines for final year medical students, facilitating the acquisition of fundamental skills that are essential for their future practice.

When implementing an ultrasound curriculum, it is important to consider several factors. One of the initial challenges is deciding which competencies should be included in the curriculum and which should not. In this case, the proposed competencies for DEGUM/EFSUMB/abdominal ultrasound were used as a basis, as they cover a range of methods and fundamental understanding of these techniques. The educational content was derived from this source, along with lessons learned from previous ultrasound projects. Unlike DEGUM ultrasound courses that include key pathologies in the foundational course, BI-POCUS focuses on practical sonography skills and fundamental information. The curriculum provides instruction on the physical fundamentals of ultrasound as well as optimizing image generation.

The most important aspect of ultrasound is the ability to perform scans, which is a fundamental requirement for producing adequate images. Therefore, the development of visual–spatial and visual-motor skills needed to operate a transducer and acquire images is crucial. These practical skills are best acquired through hands-on practice in small, supervised groups (19). It is important to introduce practical exercises as early as possible to familiarize students with different equipment and settings. Including basic ultrasound skills in medical school curricula ensures that students can perform ultrasound examinations in the early stages of their education (37). In a supervised educational environment, exercises can be practiced successfully to gain appropriate and effective experience without putting patients at risk.

Despite BI-POCUS’ focus on hands-on skills in ultrasound imaging, sufficient knowledge of the medical setting is still important. Therefore, the curriculum includes teaching of basic physical skills, anatomical knowledge, basic understanding of the pathophysiology of different pathologies, and the ability to perform professional documentation. In addition, using common and simple medical scenarios, the diagnostic value of ultrasound and its potential are highlighted, and students are taught that ultrasound enables the clinical translation of basic principles and elements of medicine. Thus, it is pivotal for educational success to combine clinical data and anatomy/pathophysiology in real time with the ultrasound image. By integrating new ultrasound skills into the examination, medical students can connect skills learned in basic science and anatomy courses with those acquired in routine clinical practice during their internship year. The advantages of POCUS are thus presented to the students in a problem-centered approach, and BI-POCUS is accompanied by subject-specific content from the individual clinics and departments. As a result, students also receive subject-specific training in their respective electives.

There are multiple factors that contribute to the quality of education. A growing consensus exists to standardize ultrasound education, establish structured clinical courses, and assess expertise according to well-defined and reproducible criteria. In this context, the equipment, the expertise of the faculty, and the stringency of the implemented structure are to be noted.

Educational techniques must be clearly defined and taught from simple to complex and then to more targeted aspects. We carefully addressed these aspects throughout the development of BI-POCUS. In the light of constantly increasing amount of knowledge a focus regarding educational goals is necessary, too (12, 22). The acquired skills are supposed to meet the requirements of daily routine, including situations in which independent work is necessary. This may include, for example, night shifts in the emergency department or on ward. Especially in the latter situation, there is often a lack of sufficient training and support from more experienced colleagues and thus a lack of feedback to allow them to learn from their own mistakes.

As part of our curriculum, students are expected to acquire the proficiency to generate precise and accurate reports of findings using the correct terminology. To achieve this, students will receive repeated structured training in the appropriate terminology under supervision, as well as independently. The implementation of standardized reporting, documentation, and interpretation of clinical consequences can contribute to a higher level of ultrasound imaging quality and comparability. A successful ultrasound curriculum should also include the circumstances in which ultrasound may or may not be the appropriate imaging modality, as well as the ability to generate sufficient images with different types of ultrasound machines.

It is essential to avoid teaching multiple skills simultaneously to avoid overburdening the learning process. Instead, it is more effective to reduce complex structures to smaller and individually taught aspects. Ultimately, the aspects taught consecutively should be connected to cement previously learned knowledge. The BI-POCUS curriculum structure ensures this by providing different levels of competence that build on each other. To consolidate the knowledge acquired, ultrasound skills should be practiced routinely throughout medical school. In addition to in-class sessions, self-training for skill development should be encouraged.

In order to optimize the hands-on experience for individual students, we made the decision to recruit instructors with varying levels of qualifications. This led to the inclusion of tutors from all three qualification levels of DEGUM, as well as peer group tutors, with the aim of minimizing group size. Our choice was based on the established effectiveness of peer teaching, which can be attributed to the lower hierarchical, social, and intellectual difference between trainees and tutors (23). However, the optimal tutor-to-student ratio for hands-on ultrasound training remains unclear, and will be evaluated within the framework of our curriculum in further studies.

Hands-on teaching is associated with higher expenses than traditional lectures and self-study, but the proper implementation of ultrasound teaching throughout medical education requires planning and resources, including capital and manpower. Considering the potential benefits of our curriculum, the additional resources required appear reasonable. Inadequate examinations by young residents can be reduced by previously acquired and consolidated ultrasound skills, leading to a reduction in patient harm. Therefore, standardized ultrasound education could potentially contribute to resolving the conflict between the quality of medical education and patient safety by guaranteeing a minimum ultrasound standard among medical students (38). Quality assurance is ensured by aligning with the standards of recognized ultrasound associations. Acquiring broad basic skills instead of immediate sub-specialization during residency allows for an interdisciplinary approach to different diseases.

Further improvement of BI-POCUS could involve video recording of ultrasound examinations to verify learning progress and the quality of the ultrasound examination. Thus, technological progress enables the processing of enormous amounts of data. This could contribute to detailed feedback with improvement proposals beyond the hectic clinical routine.

In conclusion, our study strongly emphasizes the need for ultrasound in medical education and is the first to address the various challenges of ultrasound education in a comprehensive curriculum designed to teach ultrasound imaging to students just before they begin their residency training. The BI-POCUS curriculum is not limited to a specific location, but can be universally implemented for internship year students based on international peer-reviewed quality standards. This curriculum incorporates common basic skills as well as recent innovations such as hand-held devices, providing students with a diversified ultrasound education that serves as an essential platform for further skill development during their residency. BI-POCUS offers an excellent opportunity to improve the clinical skills of young physicians.

## Data availability statement

The original contributions presented in the study are included in the article/Supplementary material, further inquiries can be directed to the corresponding author.

## Author contributions

FR, VS, WH, PB, and SP contributed to the study conception and design. Material preparation, data collection and analysis were performed by FR, VS, and SP. The first draft of the manuscript was written by FR and SP. FR, VS, WH, PB, and SP commented on previous versions of the manuscript. WH helped by manuscript editing and data collection. Manuscript editing was done by PB. All authors contributed to the article and approved the submitted version.

## Conflict of interest

The authors declare that the research was conducted in the absence of any commercial or financial relationships that could be construed as a potential conflict of interest.

## Publisher’s note

All claims expressed in this article are solely those of the authors and do not necessarily represent those of their affiliated organizations, or those of the publisher, the editors and the reviewers. Any product that may be evaluated in this article, or claim that may be made by its manufacturer, is not guaranteed or endorsed by the publisher.
