# Supplementary material for: Development and implementation of a comprehensive ultrasound curriculum for medical students: The Bonn internship point-of-care-ultrasound curriculum (BI-POCUS)
Source: Front Med (Lausanne). 2023 Mar 24;10:1072326. doi: 10.3389/fmed.2023.1072326 (PMC10080124; doi:10.3389/fmed.2023.1072326)
Supplement: Supplementary file 1 [file Table_1.docx]

| **Module 1: The general part of the curriculum I** | |
| --- | --- |
| Basics of ultrasound | Indications and medical questions |
|  | Sonographical questions |
|  | Anatomical principles |
|  | Clinical principles |
|  | Standard sections and regular scans |
|  | Pathologies |
| FAST protocol | Indications and medical questions |
|  | Sonographical questions |
|  | Anatomical principles |
|  | Clinical principles |
|  | Standard sections and regular scans |
|  | Pathologies |
|  | ABCDE scheme |
| Basics of lung  ultrasound | Indications and medical questions |
|  | Sonographical questions |
|  | Anatomical principles |
|  | Clinical principles |
|  | Standard sections and regular scans |
|  | Respiratory insufficiency |
|  | Pneumothorax |
|  | Pleural effusion, hematothorax, Pleural empyema |
|  | Peuritis |
|  | Pulmonary edema - interstitial syndrome |
|  | Pneumonia |
| Great vessels - Aorta and Vena Cava | Indications and medical questions |
|  | Sonographical questions |
|  | Anatomical principles |
|  | Clinical principles |
|  | Standard sections and regular scans |
|  | Pathologies |
| Kidney | Indications and medical questions |
|  | Sonographical questions |
|  | Anatomical principles |
|  | Clinical principles |
|  | Standard sections and regular scans |
|  | Pathologies |
| Right upper abdomen - Liver and gall bladder | Indications and medical questions |
|  | Sonographical questions |
|  | Anatomical principles |
|  | Clinical principles |
|  | Standard sections and regular scans |
|  | Pathologies |
| Left upper abdomen - Spleen | Indications and medical questions |
|  | Sonographical questions |
|  | Anatomical principles |
|  | Clinical principles |
|  | Standard sections and regular scans |
|  | Pathologies |
| **Module 2: The general part of the curriculum II** | |
| Basics of  echocardiography | Indications and medical questions |
|  | Sonographical questions |
|  | Anatomical principles |
|  | Clinical principles |
|  | Standard sections and regular scans |
|  | Pericardial effusion |
|  | Volume status |
|  | Right ventricular function |
|  | Left ventricular function |
| Pancreas | Indications and medical questions |
|  | Sonographical questions |
|  | Anatomical principles |
|  | Clinical principles |
|  | Standard sections and regular scans |
|  | Pathologies |
| Basics of intestinal  ultrasound | Indications and medical questions |
|  | Sonographical questions |
|  | Anatomical principles |
|  | Clinical principles |
|  | Standard sections and regular scans |
|  | Acute abdomen: free abdominal gas, pathological abdominal fluid, ileus. |
|  | Appendicitis and diverticulitis |
|  | Portal and mesenteric vein thrombosis |
|  | Stones: bile duct and ureter |
|  | Abdominal wall: hernia, hemorrhage. |
| Basic of Doppler US | Longitudinal, area measurements, M-Mode |
|  | Tissue and PW Doppler |
|  | CW and color Doppler |
| Extracranial brain ultrasound | Indications and medical questions |
|  | Sonographical questions |
|  | Anatomical principles |
|  | Clinical principles |
|  | Standard sections and regular scans |
|  | Pathologies |
| Trachea and thyroid | Indications and medical questions |
|  | Sonographical questions |
|  | Anatomical principles |
|  | Clinical principles |
|  | Standard sections and regular scans |
|  | Pathologies |
| DVT | (Advanced) vein US of the lower limbs |
|  | (Advanced) vein US of the upper limbs |
| Ultrasound-guided punctures | Indications and levels of difficulty |
|  | Equipment |
|  | Puncture technique |
|  | Hygiene requirement |
|  | Vascular punctures |
|  | Punctures |
| **Module 3: The subject specific part of the curriculum** | |
| Basics of obstretic and gynecological ultrasound | Indications and medical questions |
|  | Sonographical questions |
|  | Anatomical principles |
|  | Clinical principles |
|  | Standard sections and regular scans |
|  | Emergency gynecological diagnoses |
|  | Diagnostic algorithm of the gynecological emergency in the general emergency department |
|  | Sonographic findings of acute emergency diagnoses in gynecology and early pregnancy |
| Basis of muskuloskeletal ultrasound | Indications and medical questions |
|  | Sonographical questions |
|  | Anatomical principles |
|  | Clinical principles |
|  | Standard sections and regular scans |
|  | Fractures |
|  | Muscle ruptures |
|  | Tendon ruptures |
|  | Ligament ruptures |
|  | Musculoskeletal infections |
|  | Corpus alienum |
| Basics of skin ultrasound | Indications and medical questions |
|  | Sonographical questions |
|  | Anatomical principles |
|  | Clinical principles |
|  | Standard sections and regular scans |
|  | Pathologies |
| Basics of US in opthalmology | Indications and medical questions |
|  | Sonographical questions |
|  | Anatomical principles |
|  | Clinical principles |
|  | Standard sections and regular scans |
|  | Pathologies |
| Ultrasound in  intensive care unit | Doppler, cardiac physiology |
|  | Pericardial tamponade |
|  | Right ventricular dysfunction/ pulmonary hypertension |
|  | Left ventricular dysfunction, acute left heart failure with reduced (HFrEF) or preserved (HFpEF) ejection fraction |
|  | Regional wall motion abnormalities - ACS |
|  | Acute valve dysfunction, mitral regurgitation, aortic regurgitation and aortic stenosis |
|  | Aortic dissection |
| Basics of urogenital ultrasound | Indications and medical questions |
|  | Sonographical questions |
|  | Anatomical principles |
|  | Clinical principles |
|  | Standard sections and regular scans |
|  | Pathologies |
| Ultrasound of the nervous system | Indications and medical questions |
|  | Sonographical questions |
|  | Anatomical principles |
|  | Clinical principles |
|  | Standard sections and regular scans |
|  | Pathologies |
| CEUS | Technical requirements and application |
|  | Organ specific areas of application |
|  | Prospects for future areas of application |

**eTable 1: Modules associated topics**

Topics of the modules developed within the BI-POCUS curriculum
